# Next generation live-attenuated yellow fever vaccine candidate: Safety and immuno-efficacy in small animal models

**DOI:** 10.1016/j.vaccine.2021.02.033

**Published:** 2021-03-26

**Authors:** Fabienne Piras-Douce, Franck Raynal, Alix Raquin, Yves Girerd-Chambaz, Sylviane Gautheron, Martha Erika Navarro Sanchez, Manuel Vangelisti, Nathalie Mantel

**Affiliations:** a Research and External Innovation, Sanofi Pasteur, Marcy l’Etoile, France; b Analytical Sciences, Sanofi Pasteur, Marcy l’Etoile, France; c Global Project Head, Sanofi Pasteur, Marcy l’Etoile, France

**Keywords:** Yellow Fever vaccine, Neurovirulence, Neurotropism, Viscerotropism, Immunogenicity, Protection, Vero cells, Serum-free, Stamaril, YF-VAX

## Abstract

Yellow fever (YF) remains a threat to human health in tropical regions of Africa and South America. Live-attenuated YF-17D vaccines have proven to be safe and effective in protecting travellers and populations in endemic regions against YF, despite very rare severe reactions following vaccination — YF vaccine-associated viscerotropic disease (YEL-AVD) and neurological disease (YEL-AND). We describe the generation and selection of a live-attenuated YF-17D vaccine candidate and present its preclinical profile. Initially, 24 YF-17D vaccine candidate sub-strains from the Stamaril® and YF-VAX® lineage were created through transfection of viral genomic RNA into Vero cells cultured in serum-free media to produce seed lots. The clone with the ‘optimal’ preclinical profile, i.e. the lowest neurovirulence, neurotropism and viscerotropism, and immunogenicity at least comparable with Stamaril and YF-VAX in relevant animal models, was selected as the vaccine candidate and taken forward for assessment at various production stages. The ‘optimal’ vaccine candidate was obtained from the YF-VAX lineage (hence named vYF-247) and had five nucleotide differences relative to its parent, with only two changes that resulted in amino acid changes at position 480 of the envelope protein (E) (valine to leucine), and position 65 of the non-structural protein 2A (NS2A) (methionine to valine). vYF-247 was less neurovirulent in mice than Stamaril and YF-VAX irrespective of production stage. Its attenuation profile in terms of neurotropism and viscerotropism was similar to YF-VAX in A129 mice, a ‘worst case’ animal model lacking type-I IFN receptors required to initiate viral clearance. Thus, vYF-247 would not be expected to have higher rates of YEL-AVD or YEL-AND than Stamaril and YF-VAX. In hamsters, vYF-247 was immunogenic and protected against high viremia and death induced by a lethal challenge with the hamster-adapted Jimenez P10 YF virus strain. Our data suggests that vYF-247 would provide robust protection against YF disease in humans, similar to currently marketed YF vaccines.

## Highlights

• vYF-247 was cloned from YF-VAX and adapted for growth in serum-free Vero cells.
• vYF-247 selected by safety/immunogenicity/efficacy criteria in small animal models.
• vYF-247 was less neurovirulent than Stamaril and YF-VAX.
• vYF-247 had similar attenuation profile, viscerotropism, neurotropism and immunogenicity to YF-VAX.
• vYF-247 protects hamsters from lethal challenge with yellow fever Jimenez P10 virus.

## 1 Introduction

The yellow fever virus (YFV), member of the genus Flavivirus, is endemic in Africa and South America [1]. Although most YFV infections are thought to be asymptomatic [2], case-fatality among those who develop severe disease varies 20–60% depending on population (lower in Africa [~20%] than South America [40–60%]) [3]. It is possible that underlying genetic factors may determine disease mortality [4], with genetic resistance to lethal YFV infection more likely in Africans. Nonetheless, the burden yellow fever (YF) disease is predominantly in Africa; of the estimated 200,000 YF cases and 30,000 associated deaths annually, 90% occur in Africa [5].

Vaccination of YF-risk populations is the most effective means of disease management. The live-attenuated YF-17D vaccine, available since 1937, is highly efficacious and probably confers life-long immunity [6]. Over 600 million doses of this vaccine have been administered since its introduction [7]. Very rare severe reactions after vaccination, namely YF vaccine-associated viscerotropic disease (YEL-AVD) similar to disease caused by a wild-type virus infection and YF vaccine-associated neurological disease (YEL-AND) have been reported at rates of 0.004–0.8 and 0.009–0.3 per 100,000 doses distributed [8], [9], respectively. The mechanisms underlying these severe vaccine-associated events remain to be elucidated, but are generally considered related to host factors associated with immune responses to the vaccine [3], [10]. Despite these limited safety concerns, the live-attenuated YF-17D vaccine remains the mainstay of YF disease management and its overall safety profile in routine practice is favorable.

YF resurgence in some areas and the need for mass vaccination campaigns during emergency outbreak response has led to YF vaccine shortages [11]. Production capacity of live-attenuated YF-17D vaccines is limited by availability of pathogen-free embryonated hen eggs [11], [12], and the process lacks flexibility for rapid scale-up to meet short-term increased demand. These limitations can be overcome using continuous cell culture-based manufacturing processes, allowing for highly productive production with fully automated robust processes [13]. Culture-based methods also eliminate concerns about hypersensitivity reactions to egg proteins. The Vero cell line is widely used for vaccine production [14], including in the production of live-attenuated chimeric Flavivirus vaccines or vaccine candidates, as well as against YF [6], [15], [16]. Furthermore, regulatory agencies have encouraged the use of serum-free production processes in the move to reduce/eliminate use of substances of animal origin, to alleviate safety concerns about potential exposure to infectious agents, and which also allow for better lot-to-lot consistency [17].

Several animal models have been described which replicate aspects of clinical disease and have provided insights into vaccination and protection [18]. The gold standard NHP model, which serves as natural host and reservoir for YF, recapitulates key aspects of immune response to vaccination and disease progression. Small animal models that undergo clinical and pathologic changes similar to human cases have also been developed, and include A129 mice deficient for type I IFN receptors and hamsters [19], [20], [21]. Since mice are naturally resilient to infection with flaviviruses, YFV infection and replication needs to be assessed under specific conditions, for example, following direct intracerebral inoculation or in immunocompromised mice.

A129 mice with impaired type I IFN responses provides a proxy sensitive model for the characterization of YFV viscerotropism and neurotropism after subcutaneous injection and thus, can help ascertain vaccine candidate attenuation properties as per required safety criteria [19], [20]. The Syrian golden hamster (*Mesocricetus auratus*) is susceptible to viscerotropic infection with YFV derived from the Jimenez and Asibi clinical strains after serial passages in hamsters. Hence, hamsters can now be used as suitable models to evaluate YF vaccine immunogenicity, i.e. vaccine-induced functional antibody responses, as well as vaccine efficacy against lethal challenge with virulent ‘hamster-adapted’ YFV strains. These models are thus considered suitable in the evaluation of safety and immuno-protection induced by new YF vaccine candidates [6], [22].

Here, the preclinical profile of a new live-attenuated YF vaccine candidate selected and cloned from a YF-17D vaccine substrain adapted for growth in Vero cells (referred to as vYF) cultured in serum-free medium was assessed in the mouse and hamster models. As the vYF vaccine candidate was demonstrated in these small animal models to be safe and immunogenic, and to induce protection against lethal challenge, it has since progressed to clinical assessment in healthy volunteers (ClinicalTrials.gov Identifier: NCT04142086).

## 2 Material and methods

### 2.1 Vaccine strain genesis

The processes undertaken to generate homogeneous, well-defined, YF-17D virus substrains from Stamaril® and YF-VAX® vaccines adapted to grow on Vero cells in serum-free conditions, and their sequencing are summarized in detail in the supplementary information. Six vYF candidate strains that grew well on serum-free Vero cells (3 for each lineage) were selected for preclinical assessment.

### 2.2 Ethic statement

All studies with the animal models were performed in accordance with French national regulations and European Directives. Studies were conducted in animal facilities accredited by the Association for Assessment and Accreditation of Laboratory Animal Care International. The protocols were approved by the French Ministry of Research and by the committee on the ethics of animal experiments at Sanofi Pasteur, France. All efforts were made to reduce the use of animals and to minimize pain and distress. The animals could be euthanized at any time to prevent suffering if any of the following occurred: signs of suffering (cachexia, weakening, difficulty in moving or eating), parameter scores demonstrating paralysis, prostration and/or shiver, excessive body weight loss, i.e. >30% or body temperature below 32 °C.

### 2.3 Selection of vaccine candidate: neurovirulence, viscerotropism, neurotropism and immunogenicity

Initially, the six vYF candidates selected at the premaster seed lot (pMSL) stage were assessed for viscerotropism and neurotropism in A129 mice, neurovirulence in OF1 mice, and immunogenicity in hamsters in comparison with Stamaril and/or YF-VAX (as well as wild-type YFV [Asibi] in A129 mice only) as described below. The clone with the ‘optimal’ observed preclinical profile at the pMSL stage defined by a combination of several parameters (i.e. the lowest neurovirulence, neurotropism and viscerotropism, and with immunogenicity at least comparable to those induced by Stamaril and YF-VAX) was selected as the ‘optimal’ vaccine candidate, and taken forward for assessment at the master seed lot (MSL), working seed lot (WSL) and drug substances (DS) stages.

### 2.4 vYF candidates: Viscerotropism and neurotropism in A129 mice

The viscerotropism and neurotropism assessments in A129 mice were performed at Sanofi Pasteur with the vYF candidates at pMSL stage, and repeated with the ‘optimal’ vaccine candidate at MSL and WSL stages, and at CITox Lab (Evreux, France) with phase I DS. A129 mice (n = 12) aged 7–9 weeks (B&K Universal) were inoculated subcutaneously with dilution buffer or with 4 times the logarithmic value of 50% cell culture infectious dose (LogCCID_50_) of Stamaril, YF-VAX or the vYF candidate in 0.4% sodium chloride (NaCl) and 2.5% human serum albumin (HSA) diluent (vaccine candidates at pMSL stage, and ‘optimal’ vaccine candidate at MSL and WSL stages) or in a candidate stabilizing buffer (‘optimal’ vaccine candidate at phase I DS) [23]. The mice were monitored for 11 days to observe clinical signs and score their severity on a four-point scale (0 to 3) as described below. YF-NS5 qRT-PCR was used to measure the viral load in serum on Day (D) 4, D6 and D11 after inoculation, and in the brain and liver of six mice per group euthanized on D6 and D11 (supplementary materials). YF neutralizing antibodies were measured with the 50% micro-plaque reduction neutralization test (µPRNT_50_) before inoculation and on D11 post-vaccination (supplementary materials).

### 2.5 vYF candidates: Neurovirulence in OF1 mice

Neurovirulence was assessed by determining the quantity of vYF candidate estimated to produce fatal encephalitis in 50% of intracerebrally inoculated mice (mouse lethal dose 50 [MLD_50_]). The MLD_50_, described in the WHO TRS 872 (1998) Annex 2, is the historical method for titration of the viral seeds prior to cell culture methods [24]. Here, we applied the method described with an additional scoring of morbidity (clinical signs and symptoms).

These assessments were performed at Sanofi Pasteur with the vYF candidates at pMSL stage, and repeated with the ‘optimal’ vaccine candidate at the MSL and WSL stages, and at Charles River Laboratories (Les Oncins, France) with phase I DS. Female OF1 mice (Charles River Laboratories, Les Oncins, France) aged 4 weeks were injected intracerebrally under anesthesia with 0.03 mL serial dilutions (six dilutions) of vYF candidate reconstituted in 0.4% NaCl and 2.5% HSA diluent; groups of eight mice were used for each serial dilution. The mice were inoculated immediately after serial dilution reconstitution, and monitored for 21 days to observe clinical signs. The clinical signs were divided into three categories, general appearance, neurological signs, and mobility. The neurological signs were scored on a four-point scale (0 to 3) with total group scores used for the graphical representations and for between group discrimination. The four-point scale was defined as follows: 0=‘normal’, no clinical signs; 1=‘mild’ clinical signs, moving inconveniently [prancing/walking on tiptoe]; 2=‘strong’ clinical signs, partial paralysis; 3=‘prostration’, total paralysis (mice with this score were euthanized). The number of dead mice was recorded. These assessments were repeated to attain two separate independent determinations.

### 2.6 vYF candidates: immuno-efficacy in hamsters

The immuno-efficacy studies in Syrian Golden hamsters (*Mesocricetus auratus*) were performed at Sanofi Pasteur with the vYF candidates at pMSL stage, and repeated with the ‘optimal’ candidate at the MSL and WSL stages, and at Voxcan (Dommartin, France) with phase I DS. Hamsters (n = 15) aged 4–5 weeks (Janvier Labs) were inoculated subcutaneously with dilution buffer or with 5 LogCCID_50_ of Stamaril, YF-VAX or the vYF candidate diluted in 0.4% NaCl and 2.5% HSA diluent (immunogenicity evaluation of vaccine candidate at MSL and WSL stages) or in vYF stabilizing buffer (immunogenicity evaluation of phase I DS) [23]. YF neutralizing antibodies were measured with µPRNT_50_ on D26 and D44 post-vaccination (i.e. D15 post-challenge). The hamsters were challenged by the intraperitoneal (IP) route with 3.3 LogCCID_50_ of the YF Jimenez P10 strain (adapted for hamsters [21]) on D29 post-vaccination. After challenge, the hamsters were observed for clinical signs with illness scored on four-point scale for each of the three categories (general appearance, neurological signs, and mobility as described above), and temperature and weight changes recorded. The viral load was measured in serum on D5 after challenge using YF-NS5 qRT-PCR (supplementary materials).

### 2.7 Statistical analysis

For the statistical analyses, viral load and neutralizing antibody titers were Log transformed. The model’s residuals were studied to test the model’s validity (normality, extreme individuals). Heterogeneity in the results was taken into account in the model. A margin of error of 5% was used for effects of the factors.

#### 2.7.1 pMSL vaccine candidate selection

All analyses were performed on SAS v9.2® (SAS Institute, Cary, NC). An Analysis Of Variance (ANOVA) model with product as fixed factor was performed. A parametric non-inferiority test with one–sided comparison was used to compare the six vYF candidates to Stamaril. Non-parametric bilateral Wilcoxon test was used for comparisons between two doses. A Dunnett adjustment was performed for comparisons to Stamaril.

#### 2.7.2 Preclinical testing

All parametric analyses were performed on SAS v9.4®, and non-parametric analysis on EverStat6®. We wanted to compare the results obtained with the ‘optimal’ vYF candidate at different stages (WLS, MSL, DS) and evaluate the protection induced by vaccination with these products against challenge with YFV Jimenez P10.

For the mouse model irrespective of endpoint, and for viremia and neutralizing antibody titers at D26 for the hamster model, due to the non-normality of the data, adjusted non-parametric Kruskall-Wallis tests (or Wilcoxon tests comparing only two groups) were used. *Post-hoc* Bonferroni-Holm correction was applied for comparisons to a reference group (YF-VAX). For neutralizing antibody titers at D44 in the hamster model, an ANOVA1 model with product as a fixed factor was used. Comparisons to the vaccine reference (YF-VAX) were undertaken with a Dunnett adjustment.

## 3 Results

### 3.1 Selection of vYF pMSL

The nucleotide sequence and amino acid variations of the vYF pMSL candidates compared to their parental sequence are summarized in Table 1. The three clones from the Stamaril lineage had 1–5 nucleotide variations compared to the Stamaril reference sequence (Genbank JN628281.1), but none led to amino acid changes. Among the three vYF clones from the YF-VAX lineage, one (TV4221) was identical to YF-VAX reference sequence (Genbank JN628279.1) and two clones (TV3111 and TV3112) had nucleotide sequence heterogeneity that resulted in two amino acid changes. Mutation in the codon for the amino acid at position 480 of the envelope protein (E) resulted in a change from valine to leucine, and at position 65 of the non-structural protein 2A (NS2A), from methionine to valine. Two to three other nucleotide variations were identified but did not lead to amino acid changes.

**Table 1:** Nucleotide sequence and amino acid variance of the selected vYF vaccine candidate clones at the pMSL stage.

Lineage	Clone	Genome nucleotide position	Reference nucleotide	pMSL nucleotide	Variant frequency	Virus peptide	Amino acid position	Amino acid	Amino acid variant
Stamaril®	TV2212	2524	C	T	100%	NS1	24	Asp	unchanged
		4432	C	T	8%	NS2b	84	Phe	unchanged
	TV2232	5590	T	G	100%	NS3	340	Val	unchanged
		5695	C	T	100%	NS3	375	Val	unchanged
		6379	A	G	100%	NS3	603	Glu	unchanged
		7766	T	C	100%	NS5	44	Leu	unchanged
		8404	C	T	100%	NS5	256	Asp	unchanged
	TV2241	2524	C	T	100%	NS1	24	Asp	unchanged
YF-VAX®	TV3111	2411	G	T	100%	**E**	480	Val	Leu
		3701	A	G	100%	NS2a	65	Met	Val
		6496	A	G	100%	NS4a	19	Lys	unchanged
	TV3112	1408	A	T	100%	**E**	145	Val	unchanged
		2411	G	T	100%	**E**	480	Val	Leu
		3701	A	G	100%	NS2a	65	Met	Val
		6496	A	G	100%	NS4a	19	Lys	unchanged
		7027	T	C	6%	NS4b	47	Val	unchanged
	TV4221	*Identical to YF-VAX*®							

Reference corresponds to the parental sequence the clones were derived from, as indicated in the lineage column. Genome position are those of Genbank JN628281.1. Variants frequencies were calculated using the Low Frequency Variant Detection method in CLC Genomics Workbench. A 5% threshold in low frequency variant detection was applied.

No morbidity or mortality was observed in A129 mice with any of the vYF pMSL candidates (4 LogCCID_50_), except for one mouse that received the TV2232 candidate. This mouse reached the predefined endpoint and was humanely euthanized at D10 post-vaccination after >20% weight loss; this vaccine candidate was eliminated from further assessment.

**Fig. 1:**
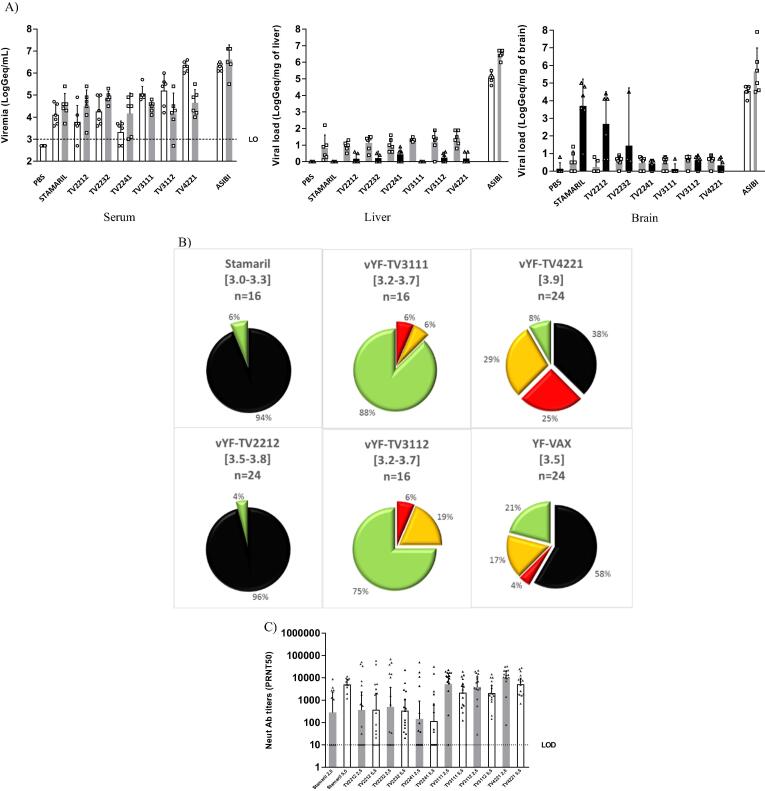
Selection of the preMaster Seed Lots (pMSL): (A) viral load measured by YF-NS5 qRT-PCR in sera, liver and brain of A129 mice inoculated at Day (D) 0 with PBS, Stamaril or with vYF vaccine candidates at pMSL stage derived from the Stamaril lineage (TV2212, TY2232 and TV2241) and YF-VAX lineage (TV3111, TV3112 and TV4221), or wild-type Asibi (all mice presented symptoms following injection with wild-type Asibi [tremor, paralysis and prostration] and 50% died) at the same dose (4 LogCCID_50_); (B) neurovirulence in OF1 mice inoculated at D0 with the vYF vaccine candidates, Stamaril or YF-VAX, at a normalized infectious titers between 3 and 3.9 LogCCID_50_/mL (neurological signs scored on a four-point severity scale as described in methods where 0 is represented as green, 1 as yellow and 2 as red, and mortality as black; aggregated maximum symptom score during the 21 days of observation presented); C) neutralizing antibody GMTs in sera collected at D26 from hamsters inoculated at D0 with 2.5 or 5.5 LogCCID_50_/dose of vYF vaccine candidates at the pMSL stage (TV2212, TV2232, TV2241, TV3111, TV3112 and TV4221) or Stamaril (D4 white, D6 grey). Individual data are also presented (open circles).

Viremia and viral loads in the liver and brain following inoculation with the vYF pMSL candidates, Stamaril, and YF wild-type virus Asibi strain in the A129 mice are depicted in Fig. 1A. All mice presented characteristic symptoms of YF viscerotropic and neurotropic disease (tremor, paralysis and prostration) following inoculation with wild-type Asibi; 50% died by D11 after inoculation, all had high viremia (GMT 6.6 LogGeq/mL at D6) and high viral loads in liver and brain (GMT 6.5 and 5.7 LogGeq/mL at D6, respectively). In contrast, no neurological symptoms and limited viremia/viral loads (except for TV4221 which induced viremia similar to wild-type virus Asibi [GMT at 6.3 LogGeq/mL]) were detected with the vYF candidates and Stamaril. The viremia achieved with the vYF candidates was not different to that induced with Stamaril (p-values > 0.2 for all vYF candidates irrespective of time point, except for TV4221). The TV4221 candidate induced higher viremia (6.3 LogGeq/mL) than Stamaril (4.1 LogGeq/mL) at D4 (p = 0.001).

Liver viral loads were generally higher at D6 than D11, irrespective of vaccine or vYF candidate assessed (Fig. 1A). At D6, liver viral loads were below 2 LogGeq/mg for all vYF candidates with no difference versus Stamaril; p-values > 0.1 at D6, with no statistical analysis on D11 due to the large number of mice below the LOD. Liver viral loads following inoculation with vYF candidates or Stamaril were at least 4 Log lower than with wild-type Asibi.

Brain viral loads following inoculation with Stamaril and vYF candidates from the Stamaril lineage ranged 1–5 LogGeq/mg (except for TV2241) at D11 post-inoculation (Fig. 1A), but remained below 1 LogGeq/mg with vYF candidates from the YF-VAX lineage. Brain viral loads with TV2212 and TV2232 were not different to those of Stamaril at D11 (p ≥ 0.06), but were higher than for the other vYF candidates (p ≤ 0.003). Of note, brain viral loads at D6 post-inoculation with the vYF candidates were at least 3 Log lower than with wild-type Asibi.

The relative neurovirulence of the vYF candidates compared to YF-VAX and Stamaril expressed as maximum neurological symptom scores during 21 days after intracerebral inoculation in OF1 mice is summarized in Fig. 1B. Due to ethical constraints on the number of mice that could be used, only 4 out of 6 clones were assessed. The Stamaril MLD_50_ titer was 6.0 LogMLD_50_/mL and death occurred in 94% of mice with normalized infectious titers 3–3.9 LogCCID_50_/mL. The neurovirulence profile of TV2212 (derived from Stamaril) was similar to that with Stamaril; neurological symptoms and death occurred in 96% of mice between D8–14 post-inoculation and the MLD_50_ titer was 6.7 LogMLD_50_/mL. YF-VAX induced neurological symptoms in 79% of mice and death in 58%, and had a MLD_50_ titer of 4.7 LogMLD_50_/mL. TV4221 induced neurological symptoms in 92% of mice and death in 38% between D9–15 post-inoculation; the MLD_50_ titer was 4.8 LogMLD_50_/mL, similar to that following YF-VAX inoculation, with the same kinetics for onset of symptoms. TV3111 and TV3112 had a different profile to YF-VAX: moderate clinical signs were observed in 12% and 25% of mice, respectively, from D1–4 before full recovery. No death was observed during the tests and as such, the MLD_50_ titer could not be calculated.

vYF candidate immunogenicity was evaluated in the hamster model based on neutralizing antibody titration as a correlate of protection. The neutralizing antibody titers against YF-17D in sera collected at D26 for the individual hamsters and group GMTs following inoculation with 2.5 or 5.5 LogCCID_50_/dose of Stamaril or vYF candidates is summarized in Fig. 1C. A marked increased neutralizing response at D26 was observed for all the immunized groups with at least 10–850-fold increase in GMTs compared to D0 (baseline). The neutralizing antibody titers were higher and more homogeneous with vYF candidates from the YF-VAX lineage (no non-responders even at the low dose, and mean titers were 10- to 100-fold higher) than those from the Stamaril lineage and Stamaril.

**Fig. 2:**
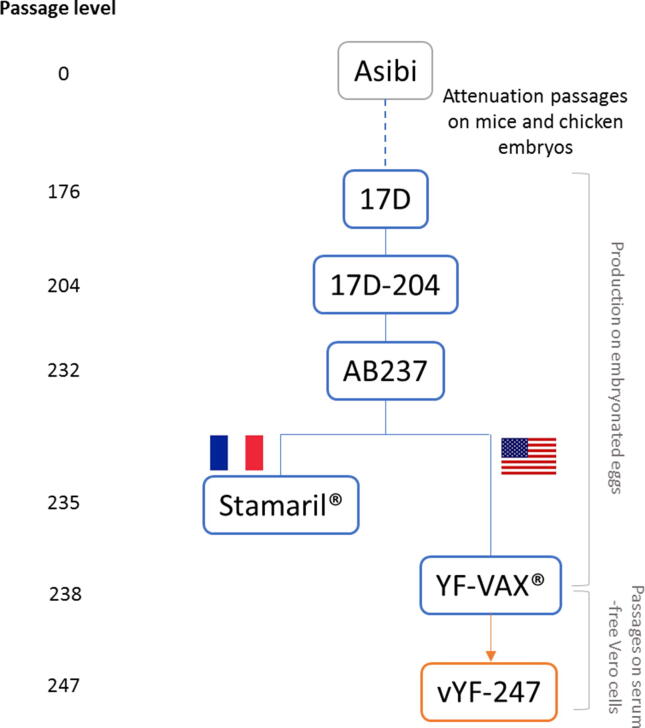
Phylogenetic tree of the vYF-247 vaccine candidate.

The vYF candidate selected for further safety, immunogenicity and protection assessments was the TV3112 clone. The other vYF candidates were eliminated as follows. TV2232 was eliminated because one A129 mouse had to be euthanized following inoculation. The other clones from the Stamaril lineage (TV2212 and TV2241) were less immunogenic than those from the YF-VAX lineage. TV4221 had an identical neurovirulence profile to YF-VAX, which is acceptable at the present time. However, TV3111 and TV3112 had the best overall profiles: very low neurovirulence, high immunogenicity and no safety signals in A129 mice. Although both TV3111 and TV3112 presented with a similar profile, we arbitrarily selected TV3112 for further assessment. MSL, WSL and DS lots from TV3112 (hence forth named vYF-247; in recognition of the number of passages undertaken from isolation of the wild-type Asibi strain [Fig. 2]) were produced from the pMSL by passages on serum-free Vero cells as follows: one passage from pMSL to MSL, one passage from MSL to WSL, and one passage from WSL to DS lot. This last passage was repeated three times to obtain the three DS lots.

### 3.2 vYF-247 safety analysis at different passages

#### 3.2.1 Neurotropism and viscerotropism assessment in A129 mice

**Fig. 3:**
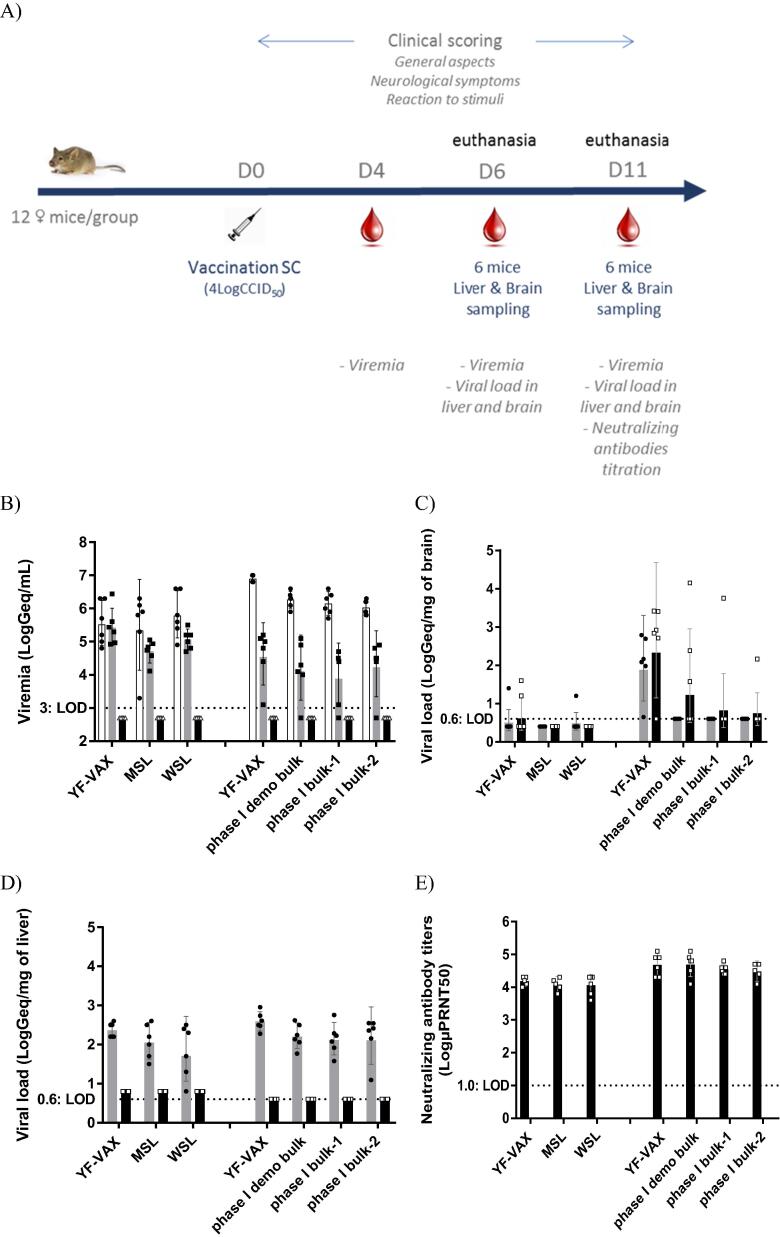
Neurotropism and viscerotropism assessments in A129 mice. The following were assessed: YF-VAX and vYF vaccine candidate at MSL and WSL stages diluted in 0.4% NaCl and 2.5% HSA or diluted in stabilization buffer for comparison of YF-VAX and vYF vaccine candidate at phase I DS bulk stage. (A) Study design; (B) Viremia; (C) Viral load in the brain; (D) Viral load in the liver; (E) Neutralizing antibody titers (Day [D] 4 white, D6 grey, D11 black). Individual data are also presented.

Fig. 3A summarizes the study protocol undertaken for the assessment of neurotropism and viscerotropism with the selected vYF-247 (TV3112) vaccine candidate in A129 mice. Viremia and viral load in brain and liver of A129 mice inoculated with 4 LogCCID_50_ of YF-VAX or vYF-247 obtained at the MSL, WSL and phase I DS stages, in two independent studies, are summarized in Fig. 3B–D. No viral RNA could be detected in the serum, brain or liver of control animals irrespective of study.

Viremia between 4.8 and 6.4 LogGeq/mL could be detected at D4 and D6 post-inoculation with YF-VAX which dropped to below the LOD by D11 (Fig. 3B). Similar viremic profiles to YF-VAX were observed at D4 and D6 post-inoculation with vYF-247 at the MSL or WSL stages (p-values > 0.8 or >0.7 for vYF-247 MSL or WSL vs YF-VAX, respectively). No difference in viremia was observed between vYF-247 at the MSL and WSL stages, irrespective of the time-point assessed (p-values > 0.09). No differences were observed between the three vYF-247 phase I DS lots (p-values > 0.3); however, these were lower than those with YF-VAX (p ≤ 0.001; 0.65–0.87 LogGeq/mL lower) at D4 post-inoculation, but were not different at D6 and D11 (p-values > 0.45).

Brain viral loads remained at or below the LOD with YF-VAX and vYF-247 at MSL and WSL stages when reconstituted in 0.4% NaCl and 2.5% HSA diluent (Fig. 3C). However, viral RNA was detected in the brain of 5/6 mice at both D6 and D11 with YF-VAX when reconstituted in stabilizing buffer that will be used to prepare the phase I vaccine in line with good manufacturing practice. The viral loads in the brain observed after immunization with the vYF-247 phase I DS were below the LOD at D6 post-immunization. At D11 post-inoculation, viral RNA was detected only in the brain of 1/6 mice in the groups immunized with vYF-247 phase I DS bulk 1 and 2, and in 3/6 mice in the group immunized with vYF-247 phase I DS demo. The viral loads in brain following inoculation with the vYF-247 phase I DS demo and bulk 1 were not significantly different from those with YF-VAX (p-values 0.37 and 0.10, respectively), but those with vYF-247 phase I DS bulk 2 (–1.79 LogGeq/mg) were significantly lower (p-values = 0.046) (Fig. 3C). There was no difference between the three phase I DS bulks (p-values > 0.5).

Liver viral loads peaked by D6 with YF-VAX and vYF-247 at MSL and WSL stages, as well as with the phase I DS bulks (Fig. 3D). Viral RNA was detected in the liver of all mice (range 0.8–3.0 LogGeq/mg), with the mean viral load at about 2 LogGeq/mg. vYF-247 at the MSL and WSL stages did not induce higher liver viral loads than YF-VAX (p-values = 1). There was no significant difference between the three vYF-247 phase I DS bulks with regard to liver viral loads (p-values > 0.2). In addition, there was no difference between vYF-247 phase I DS bulks and YF-VAX at both D6 and D11 post-inoculation (p-values > 0.05).

YF-VAX and vYF-247 at MSL and WSL stages, as well as at the phase I DS bulk stage induced similar neutralizing antibody responses in A129 mice A129 (Fig. 3E); neutralizing antibody titers achieved were far above the correlate of protection threshold (i.e. 10 µPRNT_50_), with GMTs > 12000 at D11. The neutralizing antibody titers induced by vYF-247 at the MSL or WSL stage were not lower than those induced with YF-VAX (p-values > 0.44 and > 0.51 for vYF-247 MSL and WSL vs YF-VAX, respectively). There was no significant difference in the neutralizing antibody titers induced between vYF-247 at the MSL and WSL stages (p-values > 0.26). The vYF-247 phase I DS bulks and YF-VAX elicited similar neutralizing antibody profiles in mice, with GMTs > 18000 at D11. Similarly, there were no significant differences in the neutralizing antibody GMTs between the three vYF-247 phase I DS bulks (p-values > 0.6). In addition, there was no significant difference in the neutralizing antibody GMTs of each vYF-247 phase I DS bulk and YF-VAX (p-values > 0.6).

Overall, YF-VAX and vYF-247 had similar attenuation profiles in A129 mice, and there was no difference between the vYF-247 batches irrespective of production stage.

#### 3.2.2 Neurovirulence assessment in OF1 mice

**Fig. 4:**
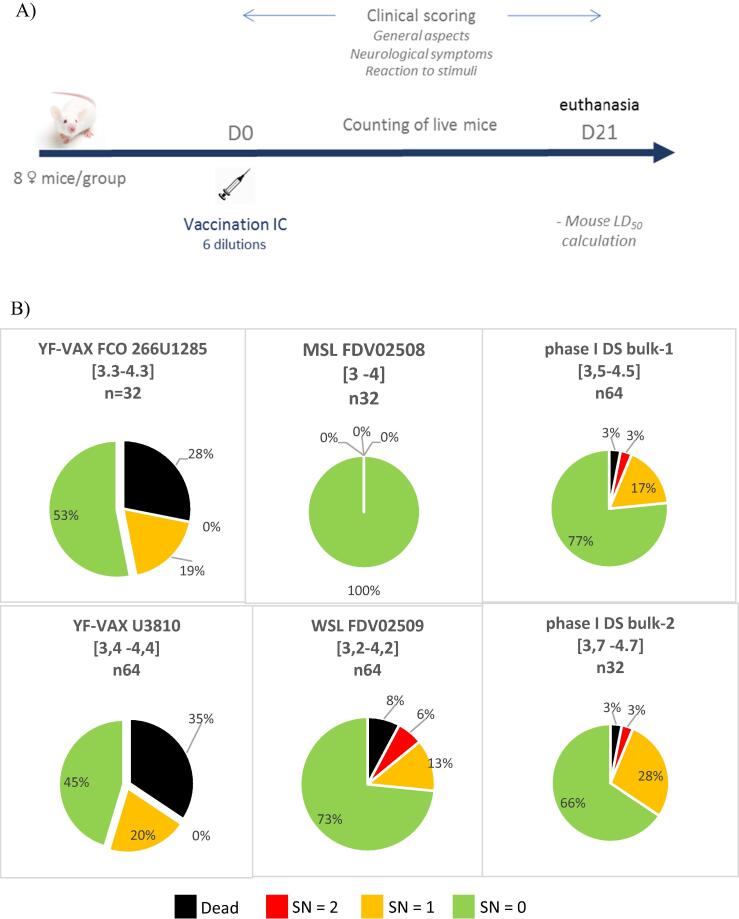
Neurovirulence assessment in OF1 mice. The following were assessed: YF-VAX and vYF-247 vaccine candidate at the MSL, WSL and phase I DS bulk stages diluted in 0.4% NaCl and 2.5% HSA at a normalized infectious titer between 3 and 4.7 LogCCID_50_/mL. (A) Study design; (B) Neurological signs scored (SN) on a four-point severity scale as described in methods where 0 is represented as green, 1 as yellow, 2 as red, and mortality as black; aggregated maximum neurological symptom score during the 21 days of observation presented.

Fig. 4A summarizes the study protocol undertaken for the assessment of neurovirulence in OF1 mice; these mice have previously been used for the evaluation of Stamaril neurovirulence at the bulk stage [25]. YF-VAX (two batches assessed) induced neurological symptoms in 52% of OF1 mice and death in 33% (both batches had similar symptoms score profiles [Fig. 4B]; aggregated maximum neurological symptom scores for both batches presented) between D9–14 post-inoculation, and had an MLD_50_ titer of approximately 3.4 LogMLD_50_/mL (least square method). In comparison, vYF-247 at the MSL stage did not induce any neurological symptoms, but induced neurological symptoms at the WSL stage in 19% of OF1 mice and death in 8% between D12–15 post-inoculation. The MLD_50_ titer for vYF-247 at the WSL stage could not be calculated (too few deaths). At the phase I DS bulk stage, the vYF-247 induced neurological symptoms in 20% to 31% of mice and death in 3% between D11–18 post-inoculation. At all stages vYF-247 was less neurovirulent than YF-VAX.

### 3.3 vYF-247 immunogenicity and protection against a lethal challenge

#### 3.3.1 Immunogenicity in hamsters

**Fig. 5:**
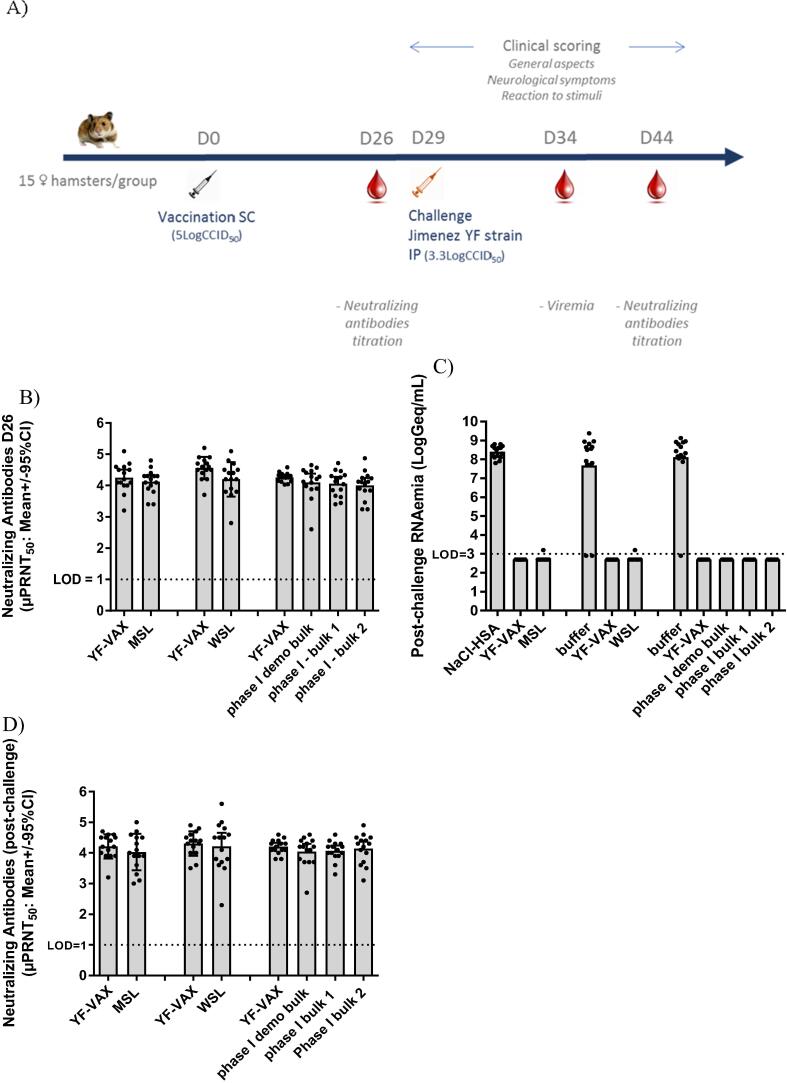
Immuno-efficacy assessments in hamsters. The following were assessed: YF-VAX and vYF-247 at the MSL stage diluted in 0.4% NaCl and 2.5% HSA; YF-VAX and vYF-247 at the WSL stage diluted in stabilization buffer; YF-VAX and vYF vaccine candidate at the phase I DS bulk stage diluted in stabilization buffer. The protection induced by the vaccines was assessed by challenge on Day (D) 29 post-vaccination with IP YF Jimenez P10 strain (3.3 LogCCID_50_) adapted for hamsters. (A) Study design; (B) Post-vaccination neutralizing antibody titers at D26; (C) Post-challenge viremia at D34; (D) Post-challenge neutralizing antibody titers at D44. Individual data are also presented.

Fig. 5A summarizes the study protocol undertaken for the assessment of immunogenicity in hamsters in three independent studies. vYF-247 at the MSL, WSL and bulk stages elicited high neutralizing responses in sera against YF-17D virus on D26 that were at least similar to that with YF-VAX (Fig. 5B). The neutralizing antibody titers achieved with YF-VAX across all hamsters (range 3.2–5.2 Log μPRNT_50_) far exceeded the threshold considered protective (i.e. 10 µPRNT_50_). None of the hamsters in the control group developed detectable neutralizing antibody responses.

The immunogenicity induced by vYF-247 at the MSL and WSL stages was not different to that of YF-VAX (p-values = 0.979 and 0.867, respectively). There was also no difference between the three vYF-247 phase I DS bulks (p-values > 0.8), or with YF-VAX (p-values ≥ 0.219). In addition, there was no difference in the immunogenicity of the vYF batches at the MSL, WSL and phase I DS bulks stages (p-value = 0.6883).

#### 3.3.2 Protection against a lethal challenge in hamsters

Hamsters were challenged on D29 with 3.3 logCCID_50_ Jimenez P10 YFV (adapted to hamsters through 10 passages) by the IP route and post-challenge mortality and morbidity were followed and viremia measured on D34 (D5 post-challenge) to assess protection. In the non-vaccinated control group, 13–14 of the 15 hamsters in each study had to be humanely euthanized or died following the challenge. These symptomatic non-vaccinated control animals had rapid changes in the clinical parameters indicative of YF pathology as soon as D4 after challenge with i) weight loss, ii) fever that peaked at D5 post-challenge, iii) clinical signs (general aspect, neurological signs and reaction to stimuli) between D4–8 post-challenge, and iv) high viremia (>7 LogGeq/mL) (Fig. 5C). Of the four hamsters that survived lethal challenge, two presented i) weight loss, ii) fever that peaked at D5 post-challenge, iii) clinical signs (general aspect, neurological signs and reaction to stimuli) between D4–8 post-challenge, and iv) high viremia (>7 LogGeq/mL) but recovered from D9 post-challenge, and the other two did not display any clinical signs nor viremia at D5 post-challenge, and had no neutralizing antibody titer at D15 post-challenge. The later animals likely did not receive the expected dose of Jimenez P10 YFV probably due to failed IP injection.

YF-VAX and vYF-247 (MSL, WSL and phase I DS bulk stages) protected against YF lethal infection. The hamsters had no morbidity symptoms or mortality (no weight loss, fever, or clinical signs) and the measured viremia levels were at or below the LOD (Fig. 5C). High neutralizing responses (range 2.3–5.6 Log µPRNT_50_) in sera against YF-17D virus were measured on D44 (D15 post-challenge) in hamsters immunized with vYF-247, similar to those achieved with YF-VAX (Fig. 5D) (no difference between groups, p-value = 0.8790). There was little difference in neutralizing antibody titers before and after the challenge in vaccinated hamsters (mean difference between D26–44 was 0.04 Log) probably indicating sterilizing immunity with high neutralizing antibody response inhibiting viral infection and spread at the time of challenge. Overall, vYF-247 across the different batches and production stages induced robust immunity and protection against YF infection similar to that with YF-VAX.

## 4 Discussion

The vYF-247 vaccine candidate was specifically selected and cloned from a YF-17D vaccine substrain adapted for growth in Vero cells cultured in serum-free medium. Compared to its parent YF-VAX strain, vYF-247 had sequence mutations in the codon for the amino acid at position 480 of the E protein and at position 65 of the NS2A protein, which resulted in amino acid changes from valine to leucine, and from methionine to valine, respectively. Interestingly, another vYF candidate (TV3111) had the same two mutations and both had similar neurovirulence and immunogenicity profiles. None of these mutations are on the known and described YF-17D attenuation-related positions [26], [27]. The mutation at position E-480 is located in the transmembrane domain of the E protein, so not accessible from the virus surface, and as such not expected to impact immunogenicity. In addition, although mutations previously described in flavivirus NS2A are related to RNA replication, virus production, virus assembly/secretion or cytopathic effects in infected cells [28], no such impact has been shown with the NS2A-65 mutation identified in our study and we did not observe a dramatic change in virus yield.

vYF-247 at all production stages was less neurovirulent than Stamaril and YF-VAX, but had similar neurotropism, viscerotropism and immunogenicity. Of note, vYF-247 did not increase viscerotropism or neurotropism compared to Stamaril and YF-VAX in the ‘worst case’ scenario, the A129 mouse model (i.e. worst case scenario as these mice lack receptors for IFN-α/β [type-I IFN receptor] required to initiate innate and adaptive immune responses involved in viral clearance [29]). A129 mice are highly susceptible to YF infection and disease, and as such represent a highly sensitive animal model for the assessment of vYF replication as well as for neurotropism and viscerotropism [19], [20]. Our comparison of Stamaril, as well as the vYF candidates, to wild-type Asibi confirms the sensitivity of this model. vYF-247 vaccine candidate appears overall to be less neurovirulent than the commercial vaccines across the small animal models assessed while maintaining similar immunogenicity, and without increasing viscerotropism. Thus, vYF-247 would not be expected to have higher safety findings or higher rates of YEL-AVD or YEL-AND than the currently marketed YF-17D vaccines. However, these incidences with the vYF-247 vaccine will not be known until it has been widely used (beyond the context of a clinical trial).

Other YF vaccine candidates produced using cell culture-based methods have included an inactivated YF-17D vaccine produced from Vero cells [6], and two live-attenuated YF-17DD vaccines produced from chicken embryo fibroblasts and skeletal muscle cells [30], [31]. The inactivated YF-17D vaccine amplified on Vero cells (though not from a biological clone as in our study) is expected to ameliorate safety concerns associated with the live-attenuated vaccine (i.e. expectation based on the absence of replication in host tissue), but has lower immunogenicity in hamsters and NHPs as well as humans based on virus equivalent dose of plaque forming units in the form of inactivated antigens relative to the live vaccine [6], [32]. In addition, the inactivated vaccine necessitates the use of an adjuvant and may require at least two injections for effective development of antibody titers. In contrast, the live-attenuated YF-17DD vaccines had similar immunogenicity to the parent YF-17DD vaccine in mice and NHPs, but also had a tendency towards higher neurovirulence than the parent strain in NHPs [31].

All vYF-247 preparations at various stages of production induced robust neutralizing antibody responses in hamsters that were at least similar to those with YF-VAX. All hamsters vaccinated with vYF-247 were protected from death and high viremia induced by lethal challenge with Jimenez P10 YFV strain (reported in the literature to induced death in about 80% of non-vaccinated hamsters [21]). Moreover, none of the hamsters that received vYF-247 displayed morbidity symptoms or viremia (near LOD at most) following lethal challenge. The absence of clinical signs and viremia in vaccinated hamsters suggests that vYF-247 induced robust protection against YF infection. Our observations with vYF-247 have now been confirmed in NHPs, and will be reported elsewhere; the NHP model is important in providing additional safety and efficacy data due to similarity with human in responses to YFV infection [18], and is an important step prior to clinical assessment.

In conclusion, the vYF-247 vaccine candidate, produced using state-of-the-art processes, without ‘additional’ product of animal or human origin in culture media, was less neurovirulent than Stamaril and YF-VAX, but had similar viscerotropism and immunogenicity. Our data suggests that vYF-247 would provide robust protection against YF infection, at least similar to that achieved with currently marketed YF-17D vaccines.

## Funding

Funding was provided by Sanofi Pasteur. The study was also partially funded by Bill and Melinda Gates Foundation grant number OPP1127586.

## Role of funding source

This study was sponsored by Sanofi Pasteur. The sponsor participated in the study design and managed all operational aspects of the study, including monitoring data collection, statistical analyses, and writing of the report.

## Contributors

FP-D, FR, AR, YG-C, SG, MENS, MV, and NM contributed to the conceptual design of the study and/or data acquisition. All authors contributed to the interpretation of the data and participated in the drafting and critical revision of this report, approved the final version and are accountable for its accuracy and integrity.

## Data Availability Statement

All data generated or analysed during this study are included in this published article (and its supplementary information files).

## Declaration of Competing Interest

The authors declare the following financial interests/personal relationships which may be considered as potential competing interests: [All authors are employees of Sanofi Pasteur and may hold shares and/or stock options in the company.].

## References

[1] World Health Organization. Yellow fever; 2019. https://www.wh.oint/news-room/fact-sheets/detail/yellow-fever [Accessed 3 February 2020].

[2] Johansson M.A., Vasconcelos P.F., Staples J.E. (2014). The whole iceberg: estimating the incidence of yellow fever virus infection from the number of severe cases. Trans R Soc Trop Med Hyg.

[3] Monath T.P., Vasconcelos P.F. (2015). Yellow fever. J Clin Virol.

[4] Blake L.E., Garcia-Blanco M.A. (2014). Human genetic variation and yellow fever mortality during 19th century U.S. epidemics. mBio.

[5] World Health Organization. Vaccines and vaccination against yellow fever. WHO position paper -- June 2013. Wkly Epidemiol Rec 2013; 88:269–83. 23909008

[6] Monath T.P., Lee C.K., Julander J.G., Brown A., Beasley D.W., Watts D.M. (2010). Inactivated yellow fever 17D vaccine: development and nonclinical safety, immunogenicity and protective activity. Vaccine.

[7] SAGE Working Group. Background Paper on Yellow Fever Vaccine; 2013. Available at: https://www.who.int/immunization/sage/meetings/2013/april/1_Background_Paper_Yellow_Fever_Vaccines.pdf [Accessed 3 Februrary 2020].

[8] Lindsey N.P., Rabe I.B., Miller E.R., Fischer M., Staples J.E. (2016). Adverse event reports following yellow fever vaccination, 2007–13. J Travel Med.

[9] Cottin P., Niedrig M., Domingo C. (2013). Safety profile of the yellow fever vaccine Stamaril(R): a 17-year review. Expert Rev Vaccines.

[10] Silva M.L., Espirito-Santo L.R., Martins M.A., Silveira-Lemos D., Peruhype-Magalhaes V., Caminha R.C. (2010). Clinical and immunological insights on severe, adverse neurotropic and viscerotropic disease following 17D yellow fever vaccination. Clin Vaccine Immunol.

[11] UNICEF. Yellow Fever Vaccine: Current Supply Outlook; 2016. Available at: https://www.unicef.org/supply/files/YF_number_3_Supply_Update.pdf [Accessed 3 Februrary 2020].

[12] Tomashek K.M., Challberg M., Nayak S.U., Schiltz H.F. (2019). Disease resurgence, production capability issues and safety concerns in the context of an aging population: Is there a need for a new yellow fever vaccine?. Vaccines.

[13] Murray J., Todd K.V., Bakre A., Orr-Burks N., Jones L., Wu W. (2017). A universal mammalian vaccine cell line substrate. PLoS ONE.

[14] Barrett P.N., Mundt W., Kistner O., Howard M.K. (2009). Vero cell platform in vaccine production: moving towards cell culture-based viral vaccines. Expert Rev Vaccines.

[15] Guy B., Saville M., Lang J. (2010). Development of Sanofi Pasteur tetravalent dengue vaccine. Hum Vaccin.

[16] Guirakhoo F., Zhang Z.X., Chambers T.J., Delagrave S., Arroyo J., Barrett A.D. (1999). Immunogenicity, genetic stability, and protective efficacy of a recombinant, chimeric yellow fever-Japanese encephalitis virus (ChimeriVax-JE) as a live, attenuated vaccine candidate against Japanese encephalitis. Virology.

[17] Hu A.Y., Tseng Y.F., Weng T.C., Liao C.C., Wu J., Chou A.H. (2011). Production of inactivated influenza H5N1 vaccines from MDCK cells in serum-free medium. PLoS ONE.

[18] Julander J.G. (2016). Animal models of yellow fever and their application in clinical research. Curr Opin Virol.

[19] Meier K.C., Gardner C.L., Khoretonenko M.V., Klimstra W.B., Ryman K.D. (2009). A mouse model for studying viscerotropic disease caused by yellow fever virus infection. PLoS Pathog.

[20] Erickson A.K., Pfeiffer J.K. (2015). Spectrum of disease outcomes in mice infected with YFV-17D. J Gen Virol.

[21] Tesh R.B., Guzman H., da Rosa A.P., Vasconcelos P.F., Dias L.B., Bunnell J.E. (2001). Experimental yellow fever virus infection in the Golden Hamster (Mesocricetus auratus). I. Virologic, biochemical, and immunologic studies. J Infect Dis.

[22] Julander J.G., Trent D.W., Monath T.P. (2011). Immune correlates of protection against yellow fever determined by passive immunization and challenge in the hamster model. Vaccine.

[23] Clenet D., Hourquet V., Woinet B., Ponceblanc H., Vangelisti M. (2019). A spray freeze dried micropellet based formulation proof-of-concept for a yellow fever vaccine candidate. Eur J Pharm Biopharm.

[24] World Health Organization. Requirments for yellow fever vaccine, Annex 2; 1998. Available at: https://www.who.int/biologicals/publications/trs/areas/vaccines/yellow_fever/en/ [Accessed 5 Februrary 2020].

[25] Barban V., Girerd Y., Aguirre M., Gulia S., Petiard F., Riou P. (2007). High stability of yellow fever 17D–204 vaccine: a 12-year restrospective analysis of large-scale production. Vaccine.

[26] Hahn C.S., Dalrymple J.M., Strauss J.H., Rice C.M. (1987). Comparison of the virulent Asibi strain of yellow fever virus with the 17D vaccine strain derived from it. Proc Natl Acad Sci USA.

[27] Beck A.S., Barrett A.D. (2015). Current status and future prospects of yellow fever vaccines. Expert Rev Vaccines.

[28] Wu R.H., Tsai M.H., Tsai K.N., Tian J.N., Wu J.S., Wu S.Y. (2017). Mutagenesis of dengue virus protein NS2A revealed a novel domain responsible for virus-induced cytopathic effect and interactions between NS2A and NS2B transmembrane segments. J Virol.

[29] Ivashkiv L.B., Donlin L.T. (2014). Regulation of type I interferon responses. Nat Rev Immunol.

[30] Souza Y.R.M., Manso P.P.A., Oliveira B.C.D., Terra M., Paschoal T., Caminha G. (2019). Generation of Yellow Fever virus vaccine in skeletal muscle cells of chicken embryos. Mem Inst Oswaldo Cruz.

[31] Freire M.S., Mann G.F., Marchevsky R.S., Yamamura A.M., Almeida L.F., Jabor A.V. (2005). Production of yellow fever 17DD vaccine virus in primary culture of chicken embryo fibroblasts: yields, thermo and genetic stability, attenuation and immunogenicity. Vaccine.

[32] Monath T.P., Fowler E., Johnson C.T., Balser J., Morin M.J., Sisti M. (2011). An inactivated cell-culture vaccine against yellow fever. N Engl J Med.

