# Supplementary material for: Next generation live-attenuated yellow fever vaccine candidate: Safety and immuno-efficacy in small animal models
Source: Vaccine. 2021 Mar 26;39(13):1846–56. doi: 10.1016/j.vaccine.2021.02.033 (PMC8047865; doi:10.1016/j.vaccine.2021.02.033)
Supplement: Supplementary data 1 [file mmc1.docx]

**Supplementary file**

**Vaccine strain genesis**

Both Stamaril^®^ and YF-VAX^®^ vaccines are based on the YF 17D-204 AB237 virus strain produced in eggs and contain highly diverse “quasi-species” populations [1]. The overall process undertaken to generate homogeneous, well-defined, YF 17D virus strains from these vaccines that were cloned and adapted to grow on Vero cells in serum-free conditions is summarized in Figure S1.

YF 17D viral RNA was obtained by purification directly from a Stamaril dose (Sanofi Pasteur) and by *in vitro* transcription from a YF-VAX infectious clone, i.e. a plasmid coding the full genome of YF-VAX (Sanofi Pasteur). Briefly, four vials of the working seed lot of the Stamaril (lot # FA238667, infectious titer 6.38 Log PFU/vial) were each suspended in RNeasy^®^ kit lysis buffer (QIAGEN^®^) and then pooled. Similarly, YF-VAX RNA obtained by *in vitro* transcription was mixed with RNase-free water and RNeasy^®^ kit lysis buffer. The RNA from both vaccines was purified by two series of extraction with phenol/chloroform/isoamyl alcohol (125:24:1; pH 4.5) and one extraction with chloroform and isoamyl alcohol (24:1) to remove all traces of phenol. The RNA was then concentrated and cleaned of any trace organic solvent by purification on silica columns with RNeasy^®^ kit (QIAGEN^®^) following manufacturer’s recommendations.

Several transfections were conducted for each RNA purification preparation with lipofectamine 2000 reagent (Life Technologies^®^) in OptiPro serum-free medium (SFM) (Life Technologies^®^) following manufacturer’s recommendation. A mixture containing the lipofectamine reagent diluted in medium and the purified RNA was added to serum-free Vero cells previously seeded in 6-well plates and extemporaneously rinsed with OptiPro SFM. The lipofectamine-RNA mixture was left in contact with the serum-free Vero cells for 4 hours at 37°C in 5% CO_2_, before addition of virus production-serum free medium (VP-SFM) to each well. Culture medium was renewed after 16 hours and then every 3 days. Transfection supernatants were collected when the cytopathic effects (cell lysis) were visible and when the genomic titer determined by YF-NS5 qRT-PCR from the culture supernatant (as described in Mantel et al. [2]) was greater than 8.0 LogGeq/mL.

After two viral amplifications on serum-free Vero cells, the viruses were used to infect serum-free Vero cells previously seeded in 6-well plates with 1.4 to 2.3 Log PFU/well. The plates were incubated for 2 hours at 37°C in 5% CO_2_ before removal of the media and addition of overlay medium containing 1% agarose. After solidification of the overlay mixture, the plates were incubated in the reverse position (lid downward) at 37°C in 5% CO_2_. As soon as cytopathic effects appeared a second overlay mixture containing 0.008% neutral red was added to each well and incubated for further 1 to 2 days under the same conditions.

Under these conditions, white spots of lysed cells (lysis plaque) rich in viruses over an otherwise red-colored cellular monolayer develop as viral particles (clones) and are released from the infected cell into their immediate surroundings. For each amplified viral dilution, two to four clones were recovered through the cover medium and suspended in VP-SFM. Each suspension was diluted in cascade steps to perform a second series of plate purification. At the end of the second cloning run, two clones per plate were again harvested; 8 clones were obtained from the Stamaril parent strain and 16 clones from the YF-VAX parent strain. Clones were amplified twice on serum-free Vero cells.

**Premaster seed lot (pMSL**) **genesis**

Viral suspensions from six vYF strains selected (selection based on their good growth on serum-free Vero cells; 3 for each lineage) were diluted in VP-SFM and added to serum-free Vero cells seeded in 175 cm^2^ flasks two days earlier at multiplicities of infection (MOI) of 0.001. The flasks were incubated for 2 hours at 37°C in 5% CO_2_ before removal of the viral inoculum and addition of fresh VP-SFM. The culture medium was renewed after 30 hours of infection. The amplified virus was harvested when the cytopathic effects were visible and the genomic titer in qRT-PCR was above 8.0 Log Geq/mL (Day 3 p.i). The supernatant was recovered and the viral suspension clarified by centrifugation; aliquots of virus were stored at <–70°C in 10% sorbitol and constituted the six clone candidate pMSLs taken for further assessment.

**Virus titration**

Viral suspensions were 4-fold serially diluted in Iscove´s modified Dulbecco´s media (IMDM, Gibco Life Technologies) with 4% fetal calf serum (FCS). Each virus dilution (150 µL) was added into 10 wells containing Vero cells that were seeded in 96-well flat bottom plates (8000 cells/well) three days earlier. After 4 days of incubation at 37°C in 5% CO_2_, supernatants were discarded and cells fixed for 15 minutes at –20°C with 85% acetone and then saturated with 2.5% milk-phosphate buffered saline (PBS)-Tween buffer solution before immunostaining with the pan-flavivirus E-specific 4G2 mouse monoclonal antibody at 2 μg/mL (hybridoma available at ATCC [cat #HB-112]). Infected foci stained with 4G2 antibody were then revealed after incubation with a Goat Anti-Mouse IgG alkaline-phosphatase conjugated antibody (CliniSciences SA, cat #1030-04) and alkaline-phosphatase substrate (BCIP/NBT, Sigma, cat# B5655). Positive wells, i.e. wells containing at least one plaque stained in black, were counted and the final titer calculated using the Least Square regression method.

**Sequencing**

Virus genomic RNA from selected clones was isolated and purified from viral stocks by silicate column isolation using the QiaAmp Viral RNA minikit (Qiagen) and amplified by RT-PCR to produce three overlapping amplicons. Pooled libraries were prepared using Nextera XT kit (Illumina), and sequenced on an Illumina Mi-Seq instrument to yield 150 base paired-end reads following suppliers’ recommendations.

Genome sequence analyses were performed using CLC Genomics Workbench (Qiagen) software suite. Paired reads were trimmed for quality and the complete genome reconstructed through assembly against the parental vaccine strain sequence from which they were derived (Stamaril^®^ or YF-VAX^®^). A variant calling analysis was also performed to identify mutations and minor variations, using the Low Frequency Variant Detection method of CLC Genomics Workbench based on the LoFreq variant detection algorithm [3]. A threshold cutoff was applied at a frequency of 5% to eliminate background noise.

**Viral load by YF-NS5 qRT-PCR**

Total RNA from serum samples was extracted using Macherey Nagel Nucleospin 96 virus kit on the Tecan EVOware platform as per manufacturer’s instruction. Total RNA from brain or liver was extracted using RNeasy kit as per manufacturer instruction. YF 17D RNA copy numbers determined by YF-NS5 qRT-PCR were based on the detection of NS5 gene fragment, as described previously [2]. The limit of detection (LOD) and limit of quantification (LOQ) of these assays were 3.0 Log genomic equivalents (GEQ)/mL (i.e. 1 to 10 plaque forming units (PFU)/mL) and 3.3 Log GEQ/mL, respectively. Samples where no virus was detected were assigned a value of 2.7 Log GEQ/mL (i.e. half the LOD) during geometric mean titer (GMT) calculations.

**Neutralization assay**

Heat-inactivated test sera starting at 1:5 dilution were two-fold serially diluted in cell culture medium (IMDM plus 4% FCS) and mixed with a challenge dose of YF 17D virus (4000 µPFU/mL) for 1 hour before inoculation into 96-well plates of confluent Vero cells. The cell monolayers were incubated for 45 hours at 37°C in 5% CO_2_. The plaques were then visualized by immunostaining with flavivirus specific monoclonal antibodies (4G2) (see details in ‘Virus titration’ section above). The neutralizing antibody titer was calculated using the least square method and corresponds to the reciprocal of the dilution demonstrating a neutralization of 50% of the plaques compared with the virus alone control wells. The LLOQ was 10.

**Figure S1: Strain selection process overview**

**Transfection** in serum-free Vero cells

**Amplifications** on serum-free Vero cells

**Viral cloning** by plaque purification

**YF-VAX® Infectious clone**

***in vitro* transcription**

**RNA Purification**

**Stamaril®**

or

**Selection of the strain**

**Supplementary file references**

[1] Beck A, Tesh RB, Wood TG, Widen SG, Ryman KD, Barrett AD. Comparison of the live attenuated yellow fever vaccine 17D-204 strain to its virulent parental strain Asibi by deep sequencing. J Infect Dis 2014;209:334-44.

[2] Mantel N, Aguirre M, Gulia S, Girerd-Chambaz Y, Colombani S, Moste C, et al. Standardized quantitative RT-PCR assays for quantitation of yellow fever and chimeric yellow fever-dengue vaccines. J Virol Methods 2008;151:40-6.

[3] Wilm A, Aw PP, Bertrand D, Yeo GH, Ong SH, Wong CH, et al. LoFreq: a sequence-quality aware, ultra-sensitive variant caller for uncovering cell-population heterogeneity from high-throughput sequencing datasets. Nucleic Acids Res 2012;40:11189-201.
